# Extraction of natural radionuclides from aqueous solutions by novel maltolate-based task-specific ionic liquids

**DOI:** 10.1007/s10967-014-3782-x

**Published:** 2014-11-27

**Authors:** Sonja Platzer, Orhan Sap, Raphlin Leyma, Gabriele Wallner, Franz Jirsa, Wolfgang Kandioller, Regina Krachler, Bernhard K. Keppler

**Affiliations:** Faculty of Chemistry, Institute of Inorganic Chemistry, University of Vienna, Waehringer Str. 42, 1090 Vienna, Austria

**Keywords:** Ionic liquids, Natural radionuclides, Extraction of radionuclides, Maltol

## Abstract

Two novel maltol-based ionic liquids, namely [A336][Mal] and [C101][Mal], were synthesized as potential extracting agents for radionuclides from water. These two room temperature task-specific ionic liquids could be easily prepared by anion metathesis starting from commercially available materials. The isolated compounds were characterized by standard analytical methods. Their application as extraction agent for U_nat._, ^234^Th, ^210^Pb, ^210^Bi, ^210^Po and ^226^Ra was elucidated by liquid–liquid extraction and scintillation counting. Uranium was totally extracted by both ionic liquids over a broad pH range (2–8), while the other radionuclides were removed with differing efficacies depending on the respective pH value.

**Electronic supplementary material:**

The online version of this article (doi:10.1007/s10967-014-3782-x) contains supplementary material, which is available to authorized users.

## Introduction

**Fig. 1:**
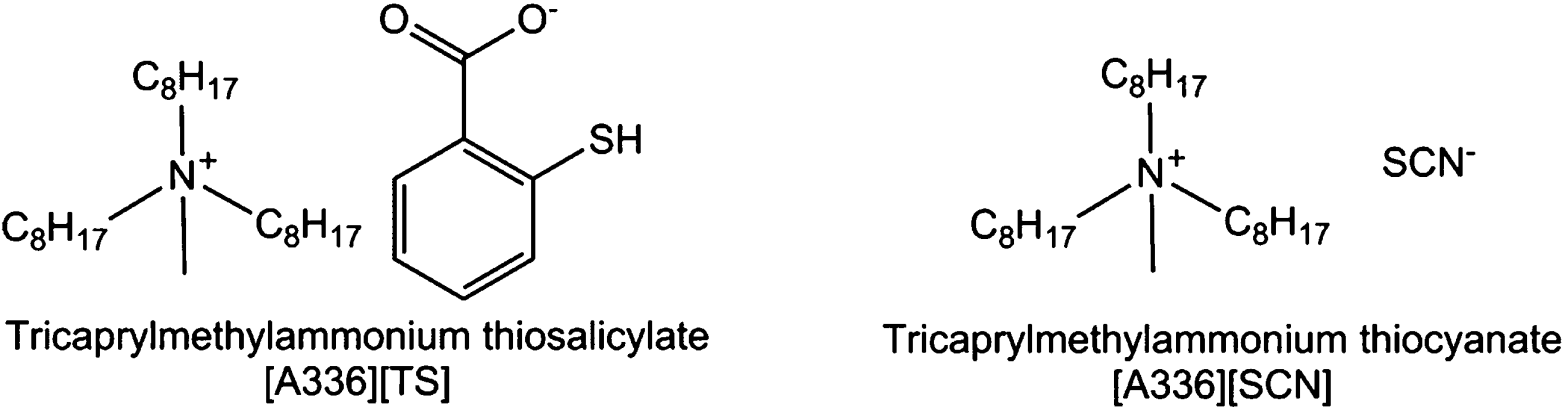
Chemical structures of [A336][TS] and [A336][SCN]

In general, ionic liquids are defined as salts with melting points below 100 °C. They are characterized by a low vapor pressure, high decomposition temperatures or electrical stability [1]. Their physicochemical properties including their hydrophobicity can be fine-tuned by specific modifications of either the cation or anion. Task-specific ionic liquids (TSILs) allow new possibilities such as heavy metal extraction by anchoring functional groups. Heavy metal ions such as Cd^2+^ and Hg^2+^ are known to be harmful and TSILs are a promising approach for the metal removal by biphasic separation. Previous liquid–liquid extraction strategies for uranium revealed high efficacies in the case of [A336][TS] and [A336][SCN] (Fig. 1) from natural mineral water. In addition, these ionic liquids were investigated for a broad range of different metal ions and promising extraction efficacies were observed for mercury and platinum [2]. Another interesting feature beside their metal extraction potential is their high distribution coefficients >1,000 compared to Ca(II) and Mg(II) [3].

Uranium is prevalent in the environment and bioavailable as uranyl ion. By the general public a few μg/day uranium are taken up by ingestion of foodstuffs and of drinking water [4]. The target organ in the human body are the kidneys and nephritis is the primary effect of uranium intoxication. Epidemiological studies in Canada, Sweden and Finland saw some minor signs of adverse renal affects but on the other hand no severe cytotoxic effects were observed even with relatively high exposures (median concentration 25 μg/L, interquartile range of 5–148 μg/L, maximum conc. of 1,500 μg/L) [5]. So the World Health Organization set a guideline value for uranium of 30 µg/L drinking water in 2012 [6]. While higher uranium levels are very rare in public water supplies, concentrations up to 1.2 mg/L of uranium were found in water samples from private wells in Europe and USA [7], [8], [9].

Aside from radiation protection issues, the recovery of uranium, thorium and lanthanides from nuclear wastes is increasingly important [10]. Recently, tricaprylmethylammonium phthalate showed promising extraction rates of U(VI), Th(IV) and Fe(III) up to 99 %. Moreover, uranium was well separated at pH 0.1 from La(III), Y(III) and Nd(III) [11]. Tetradecyltrihexylphosphonium chloride and tricaprylmethylammonium chloride are hydrophobic ionic liquids and therefore possess interesting features for metal removal by liquid–liquid extraction. Tricaprylmethylammonium chloride itself showed promising results for the extraction of ^99^Mo and ^181^W [12] while tetradecyltrihexylphosphonium chloride has been developed for the selective extraction of cobalt in the presence of nickel [13]. Maltol is in extensive research over the last decades, due to its complexation ability to a broad range of metal ions. It is used in food industry as flavour enhancer and is characterized by its preferable toxicity profile and high bioavailability. Maltol is utilized due to its highly beneficial chemical and biological properties. In addition, it is commercially available even in large scale and is well known for its synthetic versatility. Due to environmental benign character and cost efficacy, ionic liquids based on this organic scaffold might be a promising alternative to the recently reported TSIL [A336][TS] and a good starting point for the development of ionic liquids as extracting agent for uranium [14]. Previously, 4-hydroxypyrylium-based ionic liquids showed interesting features as potential media for the immobilisation of catalysts [15].

**Fig. 2:**
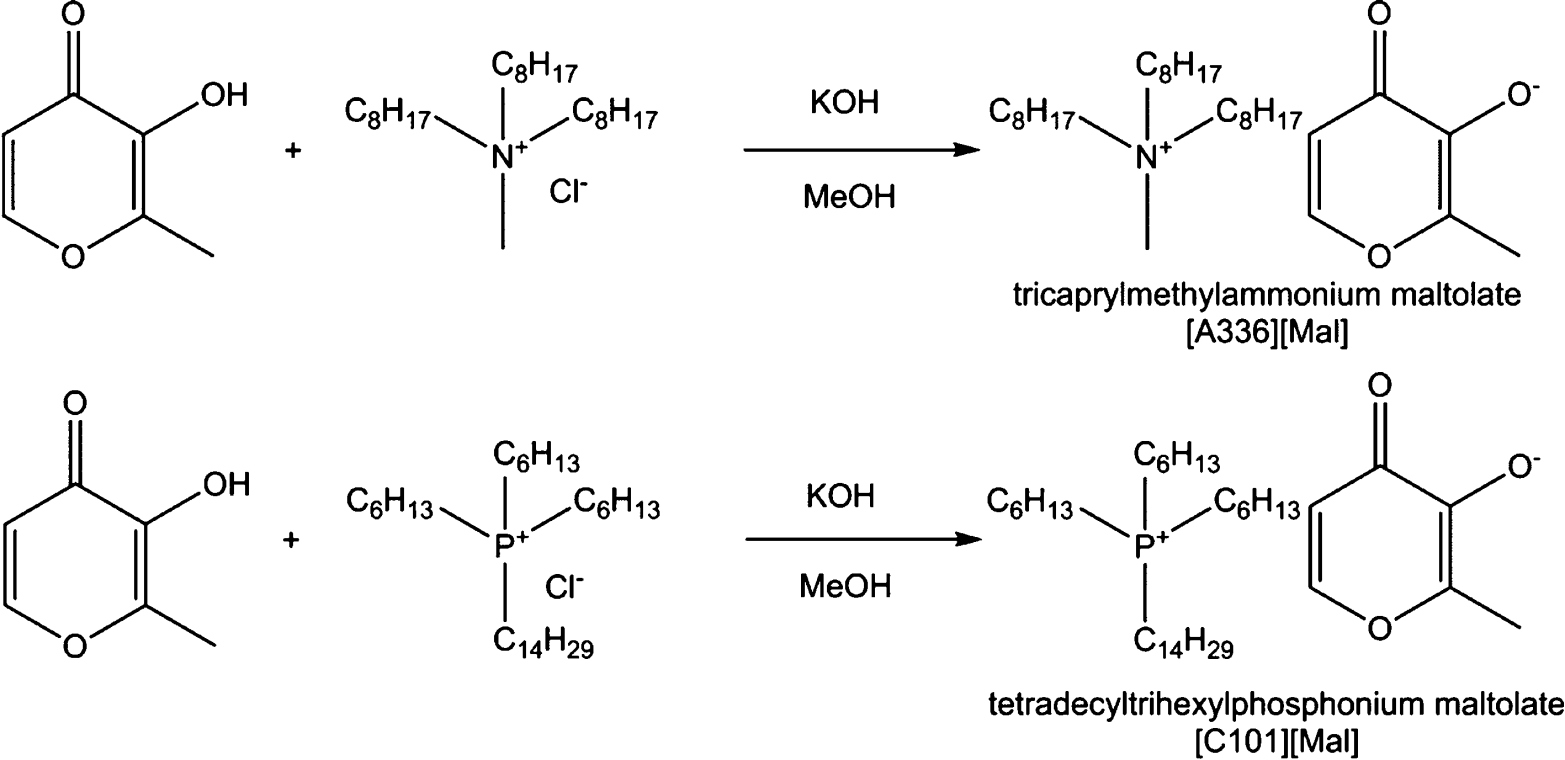
Synthetic scheme of ionic liquids [A336][Mal] and [C101][Mal]

Herein we report the synthesis of two new TSILs, tricaprylmethylammonium maltolate and tetradecyltrihexylphosphonium maltolate (Fig. 2) for the extraction of heavy metals from waste water. The presented compounds are the first step in the development of maltolato-based TSILs with optimized physicochemical parameters for the extraction of heavy metals. The respective maltolate salts were prepared by anion metathesis and characterized by standard analytical methods such as ^1^H and ^13^C NMR spectroscopy, IR and UV–vis spectroscopy. The total chloride content and the total organic carbon (TOC) values of these ionic liquids were determined. The extraction efficacies of two ionic liquids with regard to the naturally occurring radionuclides ^238^U and ^234^U, ^234^Th, ^226^Ra, ^210^Pb and its progenies ^210^Bi and ^210^Po were investigated.

## Materials and methods

3-Hydroxy-2-methyl-4(1*H*)-pyrone (maltol, 99 %) and Cyphos IL 101 (tetradecyltrihexylphosphonium chloride, 95 %) were purchased from Sigma Aldrich. Potassium hydroxide (85 %, extra pure) and Aliquat 336^®^ (mixture of quaternary ammonium compounds, tri-(C8-10)-alkylmethyl, chlorides, >75 %) were obtained from Acros. Utilized solvents were of HPLC grade and used without further purification. ^1^H and ^13^C NMR spectra were recorded on a Bruker Avance III^TM^ spectrometer in DMSO-d_6_ at 298 K using standard pulse programs at 500.10 (^1^H) and 125.76 (^13^C) MHz. UV–Vis spectra were performed on an Agilent 8453 spectrophotometer in the range of 200–1,000 nm in methanol. ATR-IR spectra were measured with a Bruker Vertex 70 Fourier transform IR spectrometer. TOC measurements were performed on a TOC-V CHP analyser (Shimadzu) and density measurements with a calibrated glass pycnometer (Klaus Hofmann). Viscosity measurements were conducted using a Kinexus rheometer (plate/plate method; temperature range: 298–323 K; test time: 2 min). The water content of [A336][Mal] and [C101][Mal] was determined utilizing a Mettler Toledo DL39 Karl Fischer (KF) coulometer; each ionic liquid was measured in triplicates. In-house standard solutions of different radionuclides were used for the extraction experiments. The uranium standard contained 27.7 μg U_nat._ in 10 μL diluted HNO_3_, corresponding to an activity of 682 mBq (^238^U and ^234^U), as well as 341 mBq ^234^Th (half-life 24.1 days) in radioactive equilibrium with ^238^U. The ^210^Pb (half-life 22.3 years) standard solution had an activity concentration of 580 mBq/10 μL. The progenies ^210^Bi (half-life 5 day) and ^210^Po (half-life 138.4 days) are in radioactive equilibrium with ^210^Pb, which means that each of them have an activity concentration of 580 mBq/10 μL. For radium extraction we used 2 mL of the reference material IAEA-431 (29.5.2001) containing 50 mBq ^226^Ra. Ingrown ^222^Rn left the sample during shaking with the IL for ^226^Ra extraction; as the activity measurements were always done immediately after the extraction step the in-growth of ^222^Rn and its short lived progenies was prevented.

## Synthesis of ionic liquids

[A336][Mal]: To a solution of maltol (100 mg, 0.79 mmol) in methanol (5 mL) potassium hydroxide (49 mg, 0.88 mmol) was added in one portion and the solution was stirred for 10 min at room temperature. Aliquat 336^®^ (106 mg, 0.26 mmol) in methanol (5 mL) was added and the reaction mixture was stirred for further 4 h. After complete evaporation, the residue was extracted with ethyl acetate/water (7 mL/4 mL). The separated organic phase was dried over Na_2_SO_4_, evaporated under reduced pressure and dried *in* *vacuo,* yielding an orange viscous oil. Yield: 122 mg (95 %). ^1^H NMR (500.10 MHz, DMSO-d_6_): δ = 7.71 (d, ^3^ *J* = 6 Hz, 1H, H6), 6.04 (d, ^3^ *J* = 6 Hz, 1H, H5) 3.15–3.25 (m, 6H, N-CH_2_), 2.96 (s, 3H, N-CH_3_), 2.19 (s, 3H, -CH_3_), 1.56–1.66 (m, 6H, -CH_2_-), 1.20–1.37 (m, 30H, -CH_2_-), 0.83–0.91 (m, 9H, -CH_3_). ^13^C{^1^H} NMR (125.76 MHz, DMSO-d_6_): δ 173.2, 155.0, 149.7, 143.6, 114.0, 61.2, 61.0, 48.0, 31.8, 31.6, 29.4, 29.3, 29.2, 28.9, 28.9, 26.3, 26.2, 22.6, 22.5, 21.8, 14.4. IR (ATR, selected bands, ν_max_): 2925, 2857, 1620, 1463, 1377, 1260, 1196, 1073, 916, 848, 661, 628 cm^−1^. UV–Vis in MeOH, λ, nm (ε, M^−1^ cm^−1^): 215 (10690), 275 (8161).

[C101][Mal]: To a solution of maltol (100 mg, 0.79 mmol) in methanol (5 mL) potassium hydroxide (49 mg, 0.88 mmol) was added in one portion and the solution was stirred for 10 min at room temperature. Tetradecyltrihexylphosphonium chloride (135 mg, 0.26 mmol) in methanol (5 mL) was added and the reaction mixture was stirred for further 4 h. After complete evaporation, the residue was extracted with ethyl acetate/water (7 mL/4 mL). The separated organic phase was dried over Na_2_SO_4_, evaporated under reduced pressure and dried *in* *vacuo,* yielding an orange viscous oil. Yield: 149 mg (94 %). ^1^H NMR (500.10 MHz, DMSO-d_6_): δ = 7.91 (d, ^3^ *J* = 6 Hz, 1H, H6), 6.21 (d, ^3^ *J* = 6 Hz, 1H, H5) 2.22 (s, 3H, -CH_3_), 2.15–2.21(m, 8H, -CH_2_-), 1.43–1.53 (m, 8H, -CH_2_-), 1.34–1.43 (m, 9H, -CH_2_-), 1.21–1.34 (m, 36H, -CH_2_-), 0.83–0.92 (m, 12H, -CH_3_). ^13^C{^1^H} NMR (125.76 MHz, DMSO-d_6_): δ 173.0, 155.1, 149.6, 143.4, 116.8, 114.0, 31.8, 30.9, 30.5, 30.4, 30.3, 30.2, 29.5, 29.5, 29.4, 29.2, 29.1, 28.6, 22.6, 22.3, 21.1, 21.0, 18.3, 18.2, 17.9, 17.8, 14.4, 14.3. IR (ATR, selected bands, ν_max_): 3251, 3061, 2923, 2854, 1653, 1629, 1458, 1369, 1255, 1223, 1197, 1077, 1022, 919, 848, 688 cm^−1^. UV–Vis in MeOH, λ, nm (ε, M^−1^ cm^−1^): 215 (16380), 277 (12392).

## Liquid–liquid extraction experiments

Extractions were performed by weighting selected amounts (100–200 mg) of ionic liquids in 15 mL polypropylene tubes and addition of about 10 mL of aqueous solution containing a known amount of U_nat._, ^210^Pb, or ^226^Ra (the pH values had been adjusted by addition of HNO_3_ or NaOH). Samples were shaken overnight at 100 rpm and subsequently centrifuged for 30 min at 3,500 rpm [the relative centrifugal force (RCF) is 1646] for phase separation. The yield of the various extractions was determined by measuring the remaining activity concentration of the respective radionuclides in the aqueous phase by liquid scintillation counting (LSC). The advantage of this method is twofold: an aliquot of the aqueous solution can be measured directly without further sample treatment, and α- and β-emitters present in the same sample can be measured simultaneously. The poor energy resolution in the α-spectrum is of no relevance, as the ^234^U/^238^U ratio is not altered by the extraction in any way.

All extraction experiments were done at least twofold with satisfying agreement. The maximum difference of the extraction yields from an aqueous solution (at a given pH value) measured for two different batches of the same IL were 20 %, comprising also the 1σ-counting uncertainties of 5–10 %. These relatively high counting uncertainties are due to the fact that high extraction yields correspond to low activities left in the aqueous phase, which is then measured. By extending the counting time the uncertainties could be reduced.

## Instrumental setup

For the LSC measurement an aliquot of the aqueous phase (usually 3 mL) was transferred to a 20 mL vial and mixed with the scintillation cocktail HiSafe III™. After cooling, the activity concentrations of the respective radionuclides were measured with a Quantulus™ 1220 low-level liquid scintillation counter (Wallac Oy, Finland, now Perkin Elmer). Counting times were 300 min or less, depending on the activity of the respective samples. As the uranium solution contains also the β-emitter ^234^Th (+ ^234^Pa), and ^210^Pb is in radioactive equilibrium with its progenies ^210^Bi (also a β-emitter) and ^210^Po (α-emitter), pulse shape analysis was used to differ between α- and β-counts (light pulses following α-emission have a longer decay time than pulses following β-emission and can therefore be distinguished electronically).

## Leaching and chloride content measurements

Leaching of the ionic liquids into the water phase was tested the following way: 100 mg of IL were shaken with double distilled water (5 mL) for 24 h. 1.2 mL of the solution were diluted with 10.8 mL double distilled water. The samples were acidified to pH 2 with HCl and purged with carrier gas for 5 min prior to combustion in the TOC analyser. The purities of the tested ionic liquids were quantified by their residual chloride ions. The chloride content was determined by dissolving the sample in 10 mL ethanol/water (1:1), acidified with HNO_3_ (half concentrated) and potentiometric titrated using AgNO_3_ as titrant based on two independent experiments.

## Results and discussion

### Synthesis and characterization

The two maltol-based ionic liquids were prepared by anion metathesis, which is a fast and sustainable standard strategy in literature [16]. Therefore, maltol was deprotonated with an excess of potassium hydroxide to ensure it reacts quantitatively and checked by ^1^H NMR. Afterwards, either Aliquat 336^®^ (a mixture of quaternary ammonium compounds) or Cyphos IL101 was added and after aqueous work up the desired products were isolated in high yield (>90 %) and sufficient purity. An excess of maltol was necessary in both syntheses due to the high solubility of maltolate in water. The isolated ionic liquids were characterized by standard analytical methods (^1^H and ^13^C NMR, infrared and UV–Vis spectroscopy, density, viscosity, water and chloride content).

Both synthesized compounds were isolated as highly viscous oils at room temperature and the formation of the 1:1 salts was confirmed by ^1^H NMR spectroscopy (Figure S1 and S2). The chloride content of the ionic liquids [A336][Mal] and [C101][Mal] has been found to be 1.5 and 1.7 %, respectively, by argentometric titration (Table 1).

**Table 1:** Physicochemical properties of [A336][Mal] and [C101][Mal]

	Leaching [mg C/L]	Cl^−^ content (%)	H_2_O content (%)	Density (298 K) [g/cm^3^]	Solubility	
					Miscible	Immiscible
[A336][Mal]	3,800	1.5	1.3	0.98	Ethyl acetate, methanol, ethanol, acetonitrile, dichloromethane, chloroform	*n*-Hexane, water
[C101][Mal]	3,470	1.7	1.3	0.98	Ethyl acetate, methanol, ethanol, acetonitrile, dichloromethane, chloroform	*n*-Hexane, water

### Physicochemical properties

**Fig. 3:**
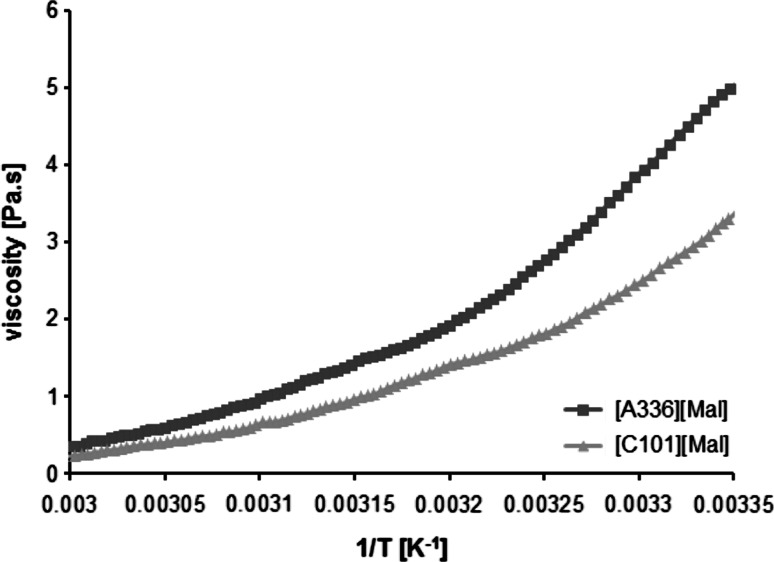
Viscosity of [A336][Mal] and [C101][Mal]

The physiochemical properties of novel ionic liquids such as melting point, density, miscibility or viscosity, define and limit the potential applications. The synthesized maltolate-based ionic liquids were found to be soluble in common organic solvents such as chloroform and ethanol, but insoluble in water. The leaching in water was determined by TOC experiments for the two ionic liquids with values of dissolved carbon of 3,800 mg/L for [A336][Mal] and 3,470 mg/L for [C101][Mal] (Table 1), respectively. This result can be explained by the high solubility of the maltolate in aqueous systems. The viscosities, determined by a temperature gradient from 298 to 323 K, are presented in Fig. 3 where [A336][Mal] shows higher viscosity than the corresponding phosphonium ionic liquid which is in good accordance to literature [17]. The different behavior might be explained by the varying chain lengths of the two applied cations. The water content of [A336][Mal] and [C101][Mal] was determined by Karl Fischer coulometry with values of 1.3 % (w/w), determined by three independent experiments. This is an important feature because water influences the physical behavior of ionic liquids [18]. Room-temperature ionic liquids can be used without further dilution for liquid–liquid extraction. It has been reported that ionic liquids consisting of quaternary ammonium and phosphonium salts do mostly have densities less than 1 g/cm^3^ [19] [20], which is in good agreement with the determined densities of [A336][Mal] and [C101][Mal] of 0.98 g/cm^3^ at 298 K (see Table 1).

### Extracting efficiency

**Fig. 4:**
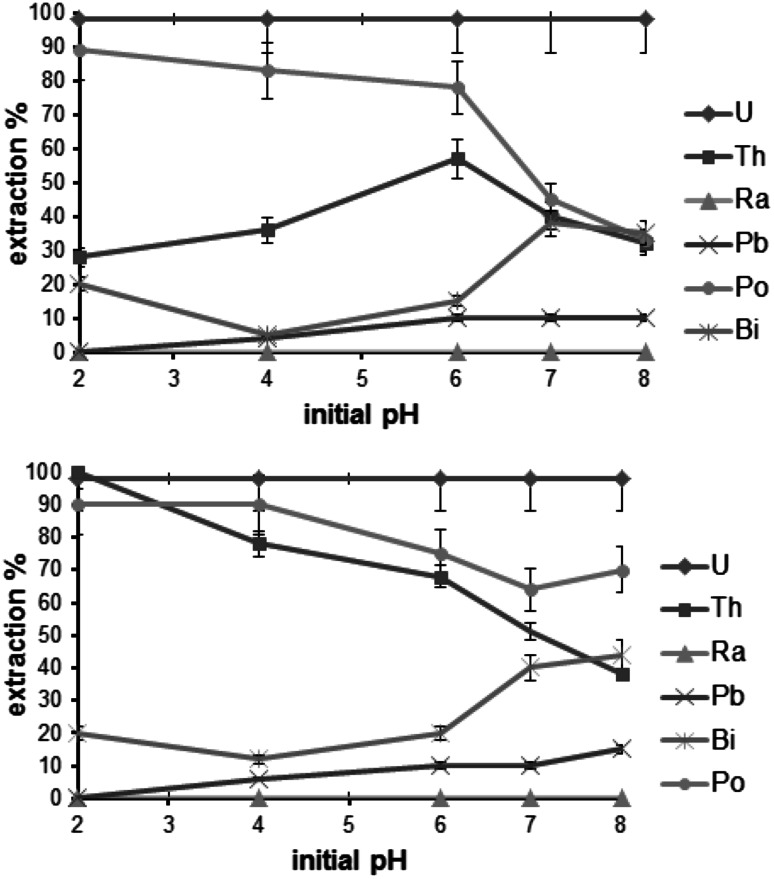
Extraction efficacy of [A336][Mal] (*top*) and [C101][Mal] (*bottom*) for different radionuclides at initial pH

Due to the known high affinity of the maltolate to a broad range of different metal ions, extraction experiments with a series of radionuclides were performed to investigate potential applications. It was found that uranium was quantitatively extracted by [A336][Mal] and [C101][Mal] (Fig. 4) and the initial pH during the experiments showed no influence on the extraction rate. This can be explained by the HSAB principle, because the uranyl cation possesses a high affinity towards the *O,O* coordination motif of the maltolate backbone. It should be kept in mind that U(VI) is present in different species depending on the pH value of the solution. For example, at pH > 2.5 UO_2_(OH)^+^, (UO_2_)_2_(OH)_2_^2+^ and (UO_2_)_3_(OH)_5_^+^ species are predominant whereas at pH < 2.5 UO_2_^2+^ is prevalent [21]. However, although the solubility of U(VI) is higher under acidic conditions, it was extracted quantitatively from all samples [21]. ^234^Th is a progeny of ^238^U and in the case of [C101][Mal] the extraction of thorium decreased at higher pH which could be explained by lower affinity of ^234^Th(OH)_2_^2+^ and other hydrolysis products to this IL compared to ^234^Th^4+^ present at lower pH values. In contrast, best extraction rate for ^234^Th with [A336][Mal] could be found at pH 6. ^210^Pb was removed less than 20 % of either [A336][Mal] or [C101][Mal] in all experiments and the pH value had no significant impact on the elimination. Up to 80 % of ^210^Po was removed at pH 2–6 with [A336][Mal], whereas [C101][Mal] was slightly less effective at pH > 4. Interestingly, at pH values ≥ 6, the extraction rate for ^210^Po decreased significantly for both compounds. Similar extraction data was observed for the ^210^Bi(III) removal of [A336][Mal] and [C101][Mal] with a maximum of about 40 % at pH 8. ^226^Ra was not extracted by both maltol-derived ionic liquids. At pH values higher than 6, only uranium could be completely eliminated from the radioactive solution.

## Conclusion

Herein, we report the straight-forward and cost-saving synthesis of two new maltol-based ionic liquids with tricaprylmethylammonium and tetradecyltrihexylphosphonium as counterions from commercially available precursors by anion metathesis. The obtained compounds were characterized by standard analytical methods and the physicochemical properties were determined. Although a high leaching of the maltol-based ionic liquids was observed, the extraction experiments with different radionuclides were very promising. The extraction of uranium showed a quantitative efficiency for [A336][Mal] and [C101][Mal] over the investigated pH range (2–8), therefore, this method represents a suitable way for uranium removal. Additionally, ^210^Po and ^234^Th were well extracted; whereas no or only low removal of either ^226^Ra, ^210^Bi or ^210^Pb was observed. Back extraction experiments with different stripping agents are ongoing.

Overall, these investigations are the first step for the development of maltol-based ionic liquids with high heavy metal extraction rates and further synthetic modifications as well as stripping experiments towards their potential applications are ongoing.

## Electronic supplementary material

Below is the link to the electronic supplementary material.

Supplementary material 1 (DOCX 222 kb)
