# Supplementary material for: Extraction of natural radionuclides from aqueous solutions by novel maltolate-based task-specific ionic liquids
Source: J Radioanal Nucl Chem. 2014 Nov 27;303(3):2483–8. doi: 10.1007/s10967-014-3782-x (PMC4514620; doi:10.1007/s10967-014-3782-x)
Supplement: Supplementary file 1 — Supplementary material 1 (DOCX 222 kb) [file 10967_2014_3782_MOESM1_ESM.docx]

**Extraction of natural radionuclides from aqueous solutions by novel maltolate-based task-specific ionic liquids**

Sonja Platzer,^a^ Orhan Sap,^a^ Raphlin Leyma,^a^ Gabriele Wallner,^a^ Franz Jirsa,^a^ Wolfgang Kandioller,^a*^ Regina Krachler,^a^ Bernhard K. Keppler^a^

^a^ Institute of Inorganic Chemistry, Faculty of Chemistry, University of Vienna, Waehringer Str. 42, A-1090 Vienna

^*^ Dr. Wolfgang Kandioller, Tel: +43-1-4277-52609, Fax: +43-1-4277-9526;

e-mail address: [wolfgang.kandioller@univie.ac.at](mailto:wolfgang.kandioller@univie.ac.at)


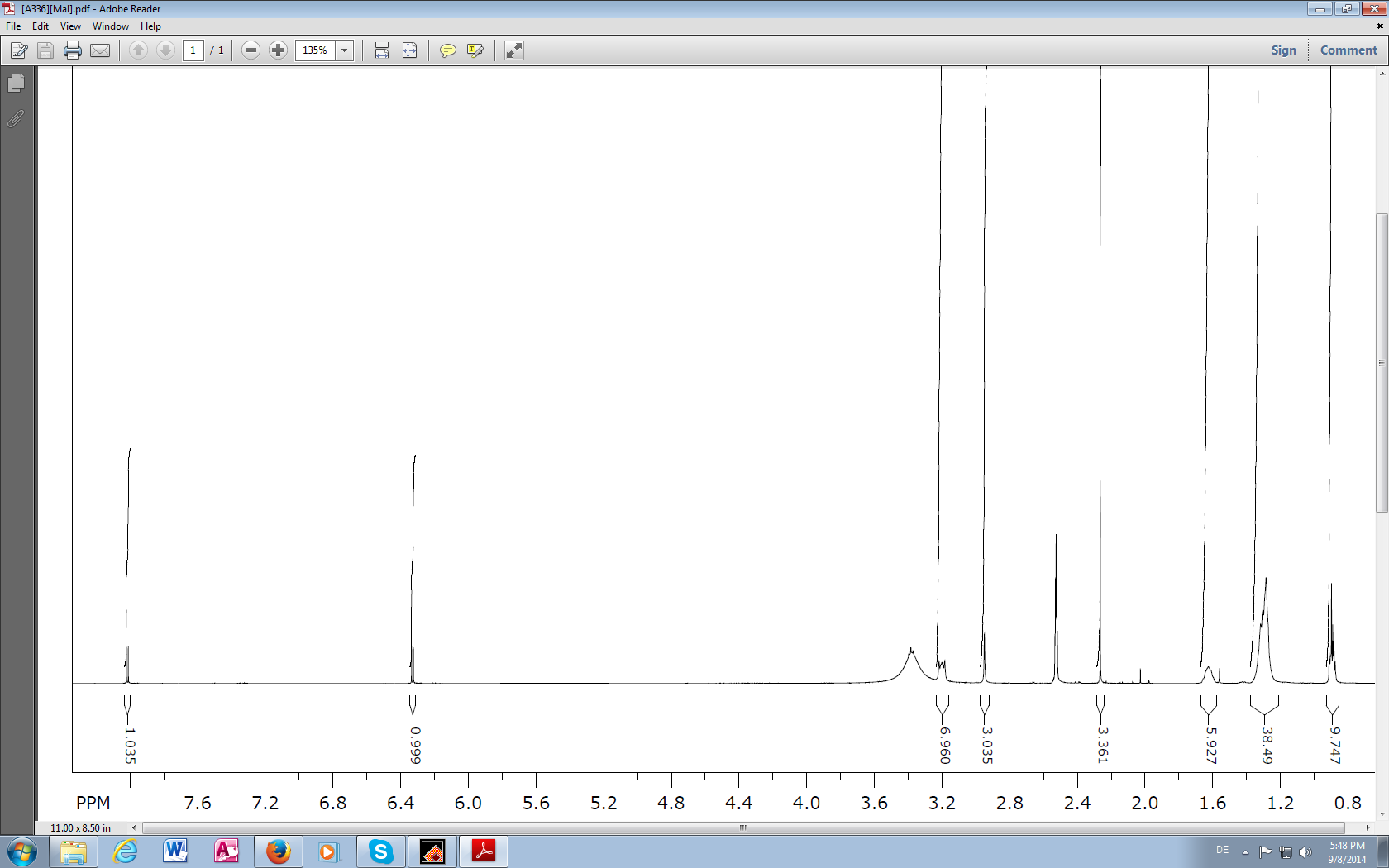


**Figure S1** ^1^H NMR of [A336][Mal]


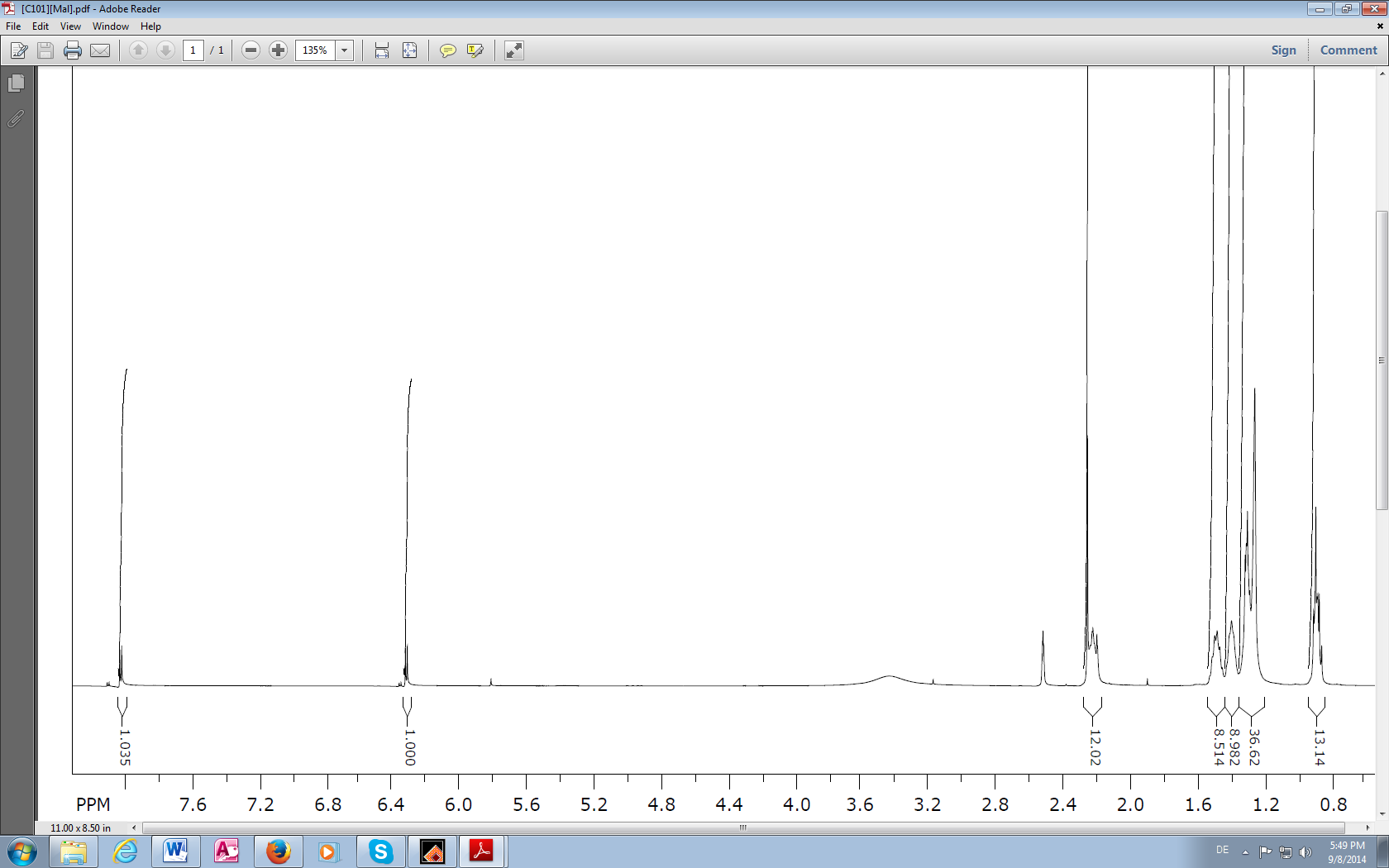


**Figure S2** ^1^H NMR of [C101][Mal]
